# SseK3 Is a *Salmonella* Effector That Binds TRIM32 and Modulates the Host’s NF-κB Signalling Activity

**DOI:** 10.1371/journal.pone.0138529

**Published:** 2015-09-22

**Authors:** Zhe Yang, Amelia Soderholm, Tania Wong Fok Lung, Cristina Giogha, Michelle M. Hill, Nathaniel F. Brown, Elizabeth Hartland, Rohan D. Teasdale

**Affiliations:** 1 Institute for Molecular Bioscience, The University of Queensland, St Lucia, Queensland, Australia; 2 Australian Infectious Diseases Research Centre, The University of Queensland, St Lucia, Queensland, Australia; 3 The University of Queensland Diamantina Institute, The University of Queensland, Translational Research Institute, Woolloongabba, Queensland, Australia; 4 Department of Microbiology and Immunology, University of Melbourne at the Peter Doherty Institute for Infection and Immunity, Melbourne, Victoria, Australia; 5 Department of Biochemistry and Molecular Biology, the University of British Columbia, Vancouver, British Columbia, Canada; Purdue University, UNITED STATES

## Abstract

*Salmonella* Typhimurium employs an array of type III secretion system effectors that facilitate intracellular survival and replication during infection. The *Salmonella* effector SseK3 was originally identified due to amino acid sequence similarity with NleB; an effector secreted by EPEC/EHEC that possesses N-acetylglucoasmine (GlcNAc) transferase activity and modifies death domain containing proteins to block extrinsic apoptosis. In this study, immunoprecipitation of SseK3 defined a novel molecular interaction between SseK3 and the host protein, TRIM32, an E3 ubiquitin ligase. The conserved DxD motif within SseK3, which is essential for the GlcNAc transferase activity of NleB, was required for TRIM32 binding and for the capacity of SseK3 to suppress TNF-stimulated activation of NF-κB pathway. However, we did not detect GlcNAc modification of TRIM32 by SseK3, nor did the SseK3-TRIM32 interaction impact on TRIM32 ubiquitination that is associated with its activation. In addition, lack of *sseK3* in *Salmonella* had no effect on production of the NF-κB dependent cytokine, IL-8, in HeLa cells even though TRIM32 knockdown suppressed TNF-induced NF-κB activity. Ectopically expressed SseK3 partially co-localises with TRIM32 at the trans-Golgi network, but SseK3 is not recruited to *Salmonella* induced vacuoles or *Salmonella* induced filaments during *Salmonella* infection. Our study has identified a novel effector-host protein interaction and suggests that SseK3 may influence NF-κB activity. However, the lack of GlcNAc modification of TRIM32 suggests that SseK3 has further, as yet unidentified, host targets.

## Introduction

The *Salmonella enterica* species are a large group of gram-negative, facultative intracellular bacteria, with most serotypes capable of causing disease in vertebrates [1]. Disease manifestation in humans ranges from gastroenteritis (caused by *S*. Typhimurium) to typhoid fever (caused by *S*. typhi) [2]. The pathogenicity of *Salmonella* and its ability to invade, avoid innate immune responses, establish an intracellular niche and subvert host nutrients to aid in bacterial survival and replication, is due to the array of effectors secreted into the cell by the type III secretion systems (T3SS) encoded by different *Salmonella* pathogenicity islands (SPI1 and SPI2) [3, 4]. Many different bacterial species responsible for enteric infections in humans often secrete homologous effectors, and the loss of certain effectors can result in partial or complete attenuation of otherwise pathogenic phenotypes. Therefore in order to better understand and treat the nature of enteric infections, it is essential to identify and characterise the function of novel *Salmonella* effectors.

The *Salmonella* secreted effector K (SseK) family members are a novel group of T3SS effectors, consisting of 3 identified members. SseK1 (previously named as *STM4157*) and SseK2 (previously named as *STM2137*) were identified due to amino acid sequence similarity with secreted proteins from attaching and effacing pathogens, such as NleB secreted by attaching and effacing pathogens such as *Citrobacter rodentium*, enteropathogenic and enterohemorrhagic *Escherichia coli* [5]. SseK1 and SseK2 are encoded in pathogenicity islets present in most *Salmonella* genomes available, and share 61% amino acid sequence homology, with most divergence occurring at their N terminal [5]. By further comparison of the genomes between *Salmonella* SL1344 and LT2 strains, SseK3 was identified as an additional member of the SseK family, which is encoded within the bacteriophage ST64B and is homologous to SseK1 and SseK2 (60% and 75% identity, respectively) [6]. A number of studies so far have examined the contribution of SseK3 to Salmonella virulence; however these studies report conflicting results. A recently published study reported a *sseK1/2/3* triple mutant strain did not attenuate *S*. Typhimurium infection in a mouse model [7], whilst another study reported the *sseK1/2/3* triple mutant attenuated *Salmonella* infection in mice using a competitive index assay [6]. Studies using RAW264.7 macrophages have also reported conflicting results, where the *sseK1/2/3* mutant is either fully virulent during infection [6, 8], or exhibits half the replication rate compared to wild-type *Salmonella* [9]. Interestingly, a recent study found *Salmonella* ΔST64B mutant has reduced survival in blood, compared to the wild-type strain [10]. Overall the findings from these studies are inconclusive, and the host targets and function of SseKs is yet to be determined. Due to sequence similarity with NleB, which blocks death receptor signaling [11, 12], we hypothesised that SseK3 may play a similar role in modulating host innate immune signaling during *Salmonella* infection.

*S*. Typhimurium secretes several anti-inflammatory T3SS effectors that target specific components of host immune signaling pathways in order to promote bacterial survival and dissemination *in vivo*. For example, GogB is an anti-inflammatory effector that inhibits expression of pro-inflammatory genes, such as IL-1, by interacting with the Skp1-Cullin1-F-box (SCF) E3 ubiquitin ligase and interfering with NF-κB activation [13]. EPEC/EHEC also inhibit NF-κB signalling and dampen host death pathways in a T3SS dependent manner with the effectors, NleB, NleE and NleC [14] [15]. NleB1 possesses N-acetylglucoasmine (GlcNAc) transferase activity and directly modifies an arginine (R) residue within the death domain containing proteins, TNFR1-associated death domain protein (TRADD) and FAS-associated death domain protein (FADD) [12]. By this mechanism, NleB1 disrupts the formation of death receptor signaling complexes, thereby blocking host death receptor signalling, in particular cell death, and promoting intestinal colonisation. Due to shared amino acid sequence similarity with NleB1, we hypothesised that the SseKs may possess a similar function as NleB1 during *Salmonella* infection [16].

In this study, we used a proteomics approach to identify host proteins targeted by SseK3 and found that SseK3 bound to the host E3 ubiquitin ligase, TRIM32. TRIM32 has previously been implicated in NF-κB signalling during UVB induced apoptosis or interferon induction for cellular antiviral response [17, 18]. Here we explored the relevance of the SseK3-TRIM32 interaction to *Salmonella* infection and in particular to the modulation of NF-κB activation.

## Materials and Methods

### Antibodies

Rabbit polyclonal anti-TRIM32 and mouse monoclonal anti-*Salmonella* Typhimurium LPS antibodies were purchased from Abcam. A mouse monoclonal anti-p230 trans Golgi antibody and a mouse monoclonal anti-early endosomal antigen 1 (EEA1) antibody were purchased from BD Transduction Laboratories. Mouse monoclonal anti-lysosomal-associated membrane protein 1 (LAMP1) marker (CD107a) was purchased from BD Pharmingen, Rabbit and mouse monoclonal anti-β-tubulin, mouse monoclonal anti-c-myc (9E10) were purchased from Sigma Aldrich. Mouse monoclonal anti-GFP antibody was from Roche. Rabbit polyclonal anti-Myc antibody was purchased from Novus Biologicals. Mouse monoclonal anti-ubiquitin antibodies were purchased from Merck-Millipore (used for immunofluorescence) and Enzo Life Sciences (used for SDS-PAGE/western blot), respectively. Mouse monoclonal anti-O-linked GlcNAc (CTD 110.6), mouse monoclonal anti-IκB, rabbit monoclonal anti-phospho NFκB p65 Ser536, rabbit monoclonal anti-NFκB p65 and rabbit polyclonal anti-GST antibodies were all from Cell Signaling Technology. Mouse monoclonal anti-His (AD1.1.10) was purchased from AbD Serotech. Rabbit polyclonal anti-GFP, Alexa Fluor 488 goat anti-mouse IgG, Alexa Fluor 546 goat anti-mouse IgG and Alex Fluor 647 goat anti-mouse IgG, Alexa Fluor 488 donkey anti-rabbit, Horse radish peroxidase (HRP) conjugated goat anti-mouse IgG and HRP conjugated goat anti-rabbit IgG were all purchased from Life Technologies. IRDye conjugated 680 anti-mouse IgG and IRDye conjugated 800 anti-mouse IgG were purchased from LI-COR Bioscience.

### Cell culture and transient transfection

A431 human epidermoid carcinoma, HeLa and HEK293 cell lines (ATCC) were maintained in Dulbecco’s modified Eagle’s medium (DMEM) (Life Technologies) supplemented with 10% (vol/vol) heat-inactivated fetal calf serum (FCS) (Life Technologies) and 2mM L-glutamine (Life Technologies) in a humidified incubator at 5% CO_2_ and 37°C. Cells were transfected with mammalian DNA constructs using Lipofectamine 2000 (Life Technologies) as per manufacturer’s instructions.

### Lentivirus production and generation of stable TRIM32 knockdown cell line

Stable knockdown cell lines were generated using lentivirus small hairpin RNA (shRNA) system (Thermo Scientific). To generate lentivirus particles, HEK293T cell were co-transfected with pGIPZ shRNA plasmids (non-silence control shRNA, RHS4346 or human TRIM32 shRNA, V3LHS_341667) together with Trans-lentiviral packaging plasmids (Thermo Scientific) using Trans-lentiviral packaging kit (Thermo Scientific) according to manufactory manual. Lentivirus particles were harvested at 48 hrs post transfection and concentrated using Lenti-X concentrator according to manufactory instruction (Clonetech).

To generate stable knockdown cell lines, HeLa cell line was transduced with lentivirus particles in serum-free medium containing 8 μg/ml of polybrene at 37°C in 5% CO_2_ for 4 hrs, when medium was replaced by normal medium for additional 24 hrs. Transduced cells were then subjected to 2 μg/ml of puromycin selection and single GFP positive cell was sorted into one well in a 96-well plate format by flow cytometry to generate monoclonal knockdown cell line.

### DNA constructs

*Mus musculus* TRIM32 cDNA (FANTOM clone ID: E430025L15) [19] was acquired from Facility for Life Science (Institute for Molecular Biosciences, The University of Queensland). Polymerase chain reaction was used to amplify the TRIM32 cDNA containing BamHI and XhoI restriction sites using high fidelity Phusion polymerase (Thermo Scientific) with the following oligonucleotides: 5’-CTAGGGATCCAGAGCAATGGCTGCGGCT-3’ (TRIM32 sense) and 5’-CTAGCTCGAGAACGAAGGAAGGAAAGGGAA-3’ (TRIM32 anti-sense) (Gene Works). TRIM32 was then subcloned in frame into pcDNA3.1 (+) vector (Invitrogen) containing a Myc epitope tag at the N-terminus by BamHI and XhoI double digestion. To generate TRIM32 containing an EGFP epitope tag at its N-terminus (GFP-TRIM32), TRIM32 was digested from Myc-TRIM32 plasmid using BamHI and XhoI double digestion, and subcloned into pEGFP-C1 (Clontech) with BglII and SalI double digestion. To generate TRIM32 construct containing a His tag at its N-terminal in a bacterial vector, TRIM32 was amplified using the following primers: 5’-CGCGGATCCATGGCTGCGGCTGCAG-3’ (sense) and 5’-CGCGAATTCTTAAGGGGTGGAATATCTTCTCAG-3’ (anti-sense) and subcloned into pET vector using BamH I and EcoRI cloning sites.

To generate the plasmid construct encoding the *Salmonella* secreted effector K 3 family member with an N-terminus GFP tag (GFP-SseK3), the wild type *sseK3* gene was amplified from *S*. Typhimurium SL1344 genomic DNA using the following primers: 5’-GAATTCATGTTTTCTCGAGTCAGAGG-3’ (sense) and 5’-GTCGACTTATCTCCAGGAGCTGATAG-3’ (anti-sense). The resultant ~1 kb PCR product was gel purified and ligated into pGEM-T-easy vector (Promega). The pGEM-T-easy construct containing the correct *sseK3* sequence was then digested with EcoRI and SalI restriction enzymes (NEB) to release the 1kb fragment which was gel purified, and subcloned into pEGFP-C2 vector (Clontech) using EcoRI and SalI restriction sites. The three catalytic active residues [12] within SseK3 were mutated into Alanine (D226A, A227A, D228A) to generate the GFP-SseK3AAA construct, using the QuikChange II Site-Directed Mutagenesis kit (Agilent Technologies). The following primers were used: 5’- CTGGAGGTGGCTGCATATATCTTGCTGCTGCTATGTTACTTACAG-3’ (sense) and reverse primer: 5’-CTGTAAGTAACATAGCAGCAGCAAGATATATGCAGCCACCTCCAG-3’ (anti-sense), and GFP-SseK3 was used as a template. All constructs were confirmed by DNA sequencing.

### Purification of recombinant GST and His tagged proteins

To purify recombinant GST tagged proteins or His tagged proteins, pGEX-NleB1 [12], pGEX-4T-1, pGEX-SseK3, pET-TRIM32 and pET-FADD [12] plasmids were transformed into BL21 strain and overnight cultures of BL21 in LB were diluted 1:100 in LB supplemented with either kanamycin or ampicillin (pGEX) with shaking to an optical density of 0.6 at 37°C. Bacterial cultures were incubated with 1 mM isopropyl-beta-D-thiogalactopyranoside (IPTG) (Sigma) and grown for a further 2 h and then pelleted by centrifugation. Proteins were purified by either nickel or glutathione affinity chromatography in accordance with the manufacturer’s instructions (Novagen). Protein concentrations were determined using a bicinchoninic acid (BCA) kit (Thermo Scientific).

### *In vitro* N-acetyl-D-glucosamine transferase assay

2 μg of purified recombinant GST or His tagged proteins were incubated either alone or in combination at 37°C for 4 h in the presence of 1 mM Uridine diphosphate N-acetylglucosamine (UDP-GlcNAc) (Sigma). Samples were boiled in SDS sample loading dye for 5 min before being subjected to SDS-PAGE and immunoblotting. Blots were probed with one of the following primary antibodies: mouse monoclonal anti-GlcNAc (1:1000), rabbit polyclonal anti-GST (1:1000) or mouse monoclonal anti-His (1:2000) antibodies, followed by incubation with horse radish peroxidase (HRP) conjugated secondary antibodies before detection with chemiluminescence (ECL) system (Thermofisher Scientific).

### Cell lysis and immunoprecipitation

A431 cells on 10 cm dishes were transfected using Lipofectamine 2000 (Life Technologies). 16 hours post transfection the cells were washed twice with cold PBS and lysed in buffer containing 50mM hydroxyethyl piperazineethanesulfonic acid (HEPES), 150mM NaCl, 1% Triton X-100, 10mM Na_4_P_2_O_7_, 30mM NaF, 1mM Na_3_VO_4_, 10mM ethylenediaminetetraacetic acid (EDTA), 0.5mM 4-benzenesulfonyl fluoride hydrochloride (AEBSF), and protease inhibitor cocktail. Lysates were centrifuged at 13 000 rpm for 10 minutes at 4°C, and the resulting supernatant was subjected to BCA assay to determine protein concentrations as per manufacturer’s instructions, by using a microplate reader (PowerWave XS, Bio-Tek) at 560nm wavelength.

To perform immunoprecipitation, equivalent amounts of protein samples were pre-cleared with 30 μl protein G conjugated agarose beads (50% slurry in PBS) for 1 hour at 4°C. The cleared supernatants were then either subjected to GFP immunoprecipitation with GFP-nanotrap beads (Protein Expression Facility, AIBN, The University of Queensland) for 1hr at 4°C, or Myc immunoprecipitation by using mouse monoclonal anti-myc antibody coupling with Protein G conjugated agarose beads overnight. The beads were then washed three times with lysis buffer and PBS, before bound proteins eluted by boiling the beads for 5 minutes in SDS-PAGE protein loading buffer.

### SDS-PAGE and immunoblotting

Equal amounts of cell lysates or immunoprecipitation samples were resuspended in SDS-PAGE protein loading buffer, before being resolved on SDS-PAGE gels. Proteins were transferred onto a PVDF membrane (Immobilon-FL, Millipore) using a semi-dry transfer apparatus (Bio-rad) under constant current (180 mA). Following, membranes were blocked with Odyssey blocking buffer for 1hr at room temperature and incubated with primary antibody in Odyssey blocking buffer containing 0.1% Tween-20/PBS (PBST) at 4 degree overnight. Membranes were washed in PBST three times for 5 minutes each wash before incubation with the appropriate IRDye conjugated secondary antibodies specified above for 1hr. Fluorescence intensities were detected by LI-COR Odyssey Infrared Imaging System (LI-COR Biosciences) [20]. For the detection of less sensitive signals, enhanced chemiluminescence (ECL) detection kit (Super Signal ECL substrate, Thermo Scientific) was used, as previously described [5]. Calculated relative molecular weight of each protein is indicated on the gels.

### NF-κB luciferase assay

HeLa cells were seeded at a concentration of 2 X 10^5^ cells/ml in 24 well plates 1 day before transfection and were 80–85% confluent at the time of transfection. 0.4 μg pEGFP-C2 or its derivatives, pEGFP-NleB1, pEGFP-NleB1AAA, pEGFP-SseK3 and pEGFP-SseK3AAA were transfected along with 0.2 μg pNF-κB-Luc (Clontech, Palo Alto CA, USA) and 0.05 μg of the control pRL-TK (Promega, Madison WI, USA). Approximately 24 h after transfection, cells were either left unstimulated or stimulated with 20 ng/ml TNF (Calbiochem, EMD4Biosciences) for 16h. The cells were harvested and analysed by the dual luciferase reporter assay according to the manufacturer’s instructions (Promega). Briefly, cells from each well were washed once with PBS and lysed in 100 μl of 1X passive lysis buffer. Cell lysates were centrifuged at full speed for 2 mins and 20 μl of each centrifuged cell lysate were loaded in a 96 well plate. First, 50 μl of Luciferase Assay Reagent II (Promega) was added to the lysates for firefly luciferase activity measurement using a luminescence optic of the FluoStar Omega plate reader (BMG LABTECH), and then 50 μl of 1X Stop & Glo Reagent (Promega) was added to the lysates to quench the firefly luciferase activity and activate the *Renilla* luciferase activity for measurement. The firefly luciferase activity of each sample was normalized to the *Renilla* luciferase activity.

To determine the effect of TRIM32 knockdown on TNF stimulated NFκB activity. Stable HeLa non-silence GFP and HeLa TRIM32 knockdown cell lines were subjected to NF-κB luciferase assay as described above.

### Indirect immunofluorescence microscopy

Indirect immunofluorescence microscopy was performed using standard procedures as described previously [21]. A431 cells were fixed in 4% paraformaldehyde in PBS for 20 minutes, before being permeabilised in 0.1% Triton X-100 in PBS for 10 minutes. After blocking with 2% BSA in PBS for 30 minutes to minimise non-specific binding, cells were labelled with the primary antibodies against p230, EEA1, Myc tag or ubiquitin for 1 hour at room temperature, followed by washing in 2% BSA in PBS 5 times before the incubation with Alexa Fluor conjugated secondary antibodies. Coverslips were washed with 2% BSA in PBS and mounted onto glass slides using Dako Fluorescent Mounting Medium. Images were captured using a confocal laser scanning microscope (LSM 510 Meta, Zeiss) under 63x Oil DIC objective. The images and co-localisation analysis were processed using ImageJ 1.46c software.

To determine the co-localisation of GFP-SseK3 to individual organelle markers, each image containing two channels: far-red channel for organelle markers (p230, EEA1 and LAMP1), and GFP channel for GFP-SseK3 expression, was segmented into individual channels and the fluorescent signal for each channel was compared to a predetermined threshold. Pearson’s co-efficient was then measured, and the total co-localisation between intracellular markers for each cell was recorded for each image. Co-localisation was also measured between endogenous ubiquitin, GFP-SseK3 and Myc-TRIM32 using Pearson’s co-efficient.

### *Salmonella* infection

Cultures for wild type *Salmonella* Typhimurium strain SL1334, ΔinvA (SPI 1 mutant), ΔssaR (SPI 2 mutant), ΔsseK1, ΔsseK2, Δssek3 and ΔsseK123 invA were described previously [5], [22]. Bacterial cultures were routinely maintained on Luria-Bertani agar plates containing 100μg/ml of ampicillin.

*Salmonella* infection assay with gentamicin protection was conducted [23]. The day prior to infection, 2 x 10^4^ A431 cells were seeded on glass coverslips in 24 well plate format with serum containing DMEM. On the day of infection, overnight cultures of wild type SL1344 expressing RFP were diluted 1:33 in Luria-Bertani LB broth, and grown with aeration for an additional 3 hours at 37°C to late log phase growth. The *Salmonella* culture was then pelleted, washed and resuspended in PBS, and A431 cells were infected at a multiplicity of infection (MOI) of 1. Thirty minutes post infection cells were washed with serum containing DMEM and incubated in media supplemented with 100 μg/ml gentamicin for one hour, followed by maintaining in media containing 10 μg/ml gentamicin. At 0, 0.5, 1, 2, 4, 8 and 20 h post infection, cells were fixed in 4% PFA for 20 min and indirect immunofluorescence was performed as described above.

### Production of IL-8 during *Salmonella* infection

HeLa cells were seeded at a concentration of 2 X 10^5^ cells/ml 2 days before infection. *Salmonella* strains were first inoculated in LB broths containing appropriate antibiotic selection and grown overnight with shaking at 37°C. Overnight cultures were then subinoculated 1/200 in fresh LB broths and grown under static conditions for additional 20 hrs at 37°C. HeLa cells were then infected at a MOI of 50 for 1 h, followed by replacing with media containing 100 μg/ml gentamycin for 1 h. HeLa cells were then further grown in the media containing 10 μg/ml gentamycin with untreated alone or the stimulation by 20 ng/ml TNFα for 6 hrs. The supernatants were collected to measure IL-8 secretion using the Quantikine Human IL-8 Immunoassay (R&D Systems, MN) according to the manufacturer’s instructions. Differences in IL-8 secretion were assessed for significance by one-way analysis of variance (ANOVA) with Turkey’s Multiple Comparison post-test.

## Results and Discussion

### Ectopically expressed SseK3 interacts with TRIM32

SseK3 is a SPI2 effector secreted into host cells during *Salmonella* infection [5]. To determine the host proteins that interact with SseK3, N-terminal GFP tagged SseK3 (GFP-SseK3) was transiently transfected into HEK293 cells and cell lysates precipitated with GFP-nanotrap agarose beads. Untransfected and EGFP expressing HEK293 cells were used as negative controls to identify non-specific binding partners. Precipitates were separated on SDS-PAGE and stained with Colloidal Coomassie Blue (S1 Fig). Protein bands corresponding to GFP-SseK3 binding proteins were excised digested with trypsin and identified by HPLC-ESI-MS/MS and database searching as previously described [24] [25]. Of the proteins identified that co-precipitated with SseK3, 18 unique peptides of Tripartate Motif 32 (TRIM32) were identified from SseK3 immunoprecipitated samples that were not identified in the samples from untransfected cells or EGFP expressing cells. TRIM32 is an E3 ubiquitin ligase involved in protein ubiquitination. Ubiquitination is a multi-step post-translational modification involving the attachment of ubiquitin to proteins, which may target the protein for degradation by the proteasome or regulate protein activity and subcellular localisation [26, 27]. TRIM32 is important in regulating the innate antiviral response against DNA and RNA viruses [18], suggesting that it may play a role in mounting a pro-inflammatory response during infection with invading pathogens. Hence the interaction of SseK3 with TRIM32 was of interest as it may contribute to *Salmonella* avoidance of innate immune activation during intracellular infection.

**Fig 1:**
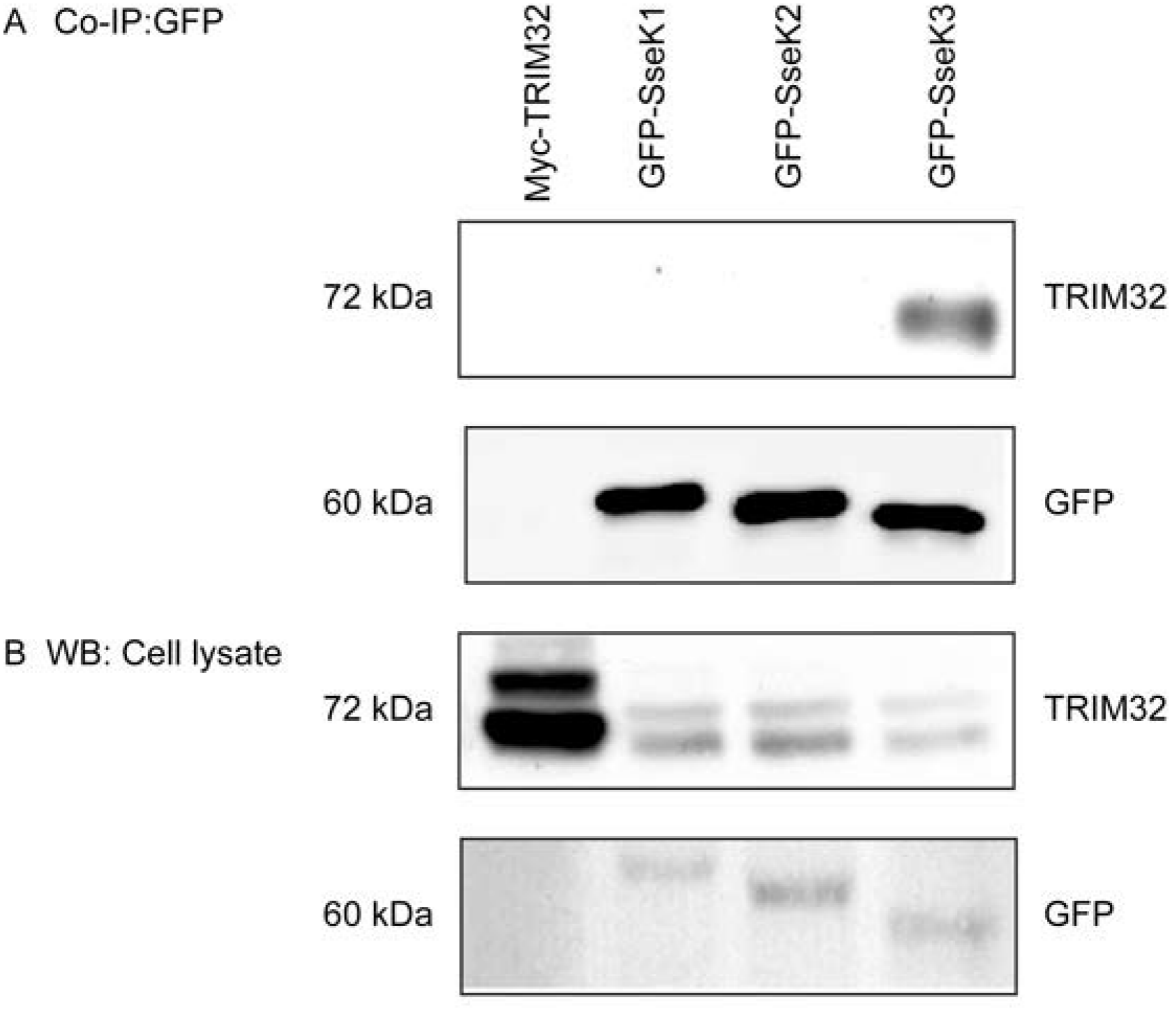
Endogenous TRIM32 interacts with GFP-SseK3. Sub-confluent A431 cells were transiently transfected with plasmids encoding Myc-TRIM32, GFP-SseK1, GFP-SseK2 or GFP-SseK3. 16–18 h post transfection, cells were washed with chilled PBS and lysed on ice using TK lysis buffer. Equal amount of cell lysates were used for the immunoprecipitation using GFP-nanotrap beads. Immunoprecipitated complexes **(A)** and cell lysates **(B)** were resuspended in SDS sample loading buffer and boiled for 5 min before resolved by SDS-PAGE, and transferred onto Immobilon-P membrane. Membranes were incubated with primary antibodies against GFP and TRIM32, followed by HRP conjugated secondary antibodies and detected by enhanced chemiluminescence (ECL) system. Untransfected cells were used as a negative control. Representative images from three independent experiments are shown.

SseK3 is related to two other T3SS effectors produced by *Salmonella* and secreted into the host cytoplasm, SseK1 and SseK2. To confirm if TRIM32 interacted selectively with SseK3, plasmids encoding Myc tagged TRIM32 (Myc-TRIM32) alone, GFP-SseK1, GFP-SseK2 or GFP-SseK3 were transfected into A431 cells and immunoprecipitation was performed. The GFP-fusion proteins were isolated from cell lysates using GFP-nanotrap beads, followed by SDS-PAGE and immunoblotting to detect TRIM32 (Fig 1). Myc-TRIM32 transfected cells were used as the negative control for GFP immunoprecipitation and the positive control for TRIM32 antibody. GFP-SseK1, GFP-SseK2 and GFP-SseK3 were precipitated in a similar efficiency by GFP-nanotrap beads (Fig 1). From the precipitated samples, TRIM32 co-immunoprecipitated exclusively with GFP-SseK3 (Fig 1). Endogenous TRIM32 is known to exist as 72 kDa in a non-ubiquitinated form and 80 kDa as a monoubiquitinated form as well as higher molecular weight forms corresponding to polyubiquitinated forms [28]. From cell lysates, both non-ubiquitinated and mono-ubiquitinated forms of TRIM32 were detected endogenously or when overexpressed (Fig 1). Interestingly, SseK3 mainly bound to the 72 kDa non-ubiquitinated form, but not the 80 kDa mono-ubiquitinated form of TRIM32 (Fig 1).

**Fig 2:**
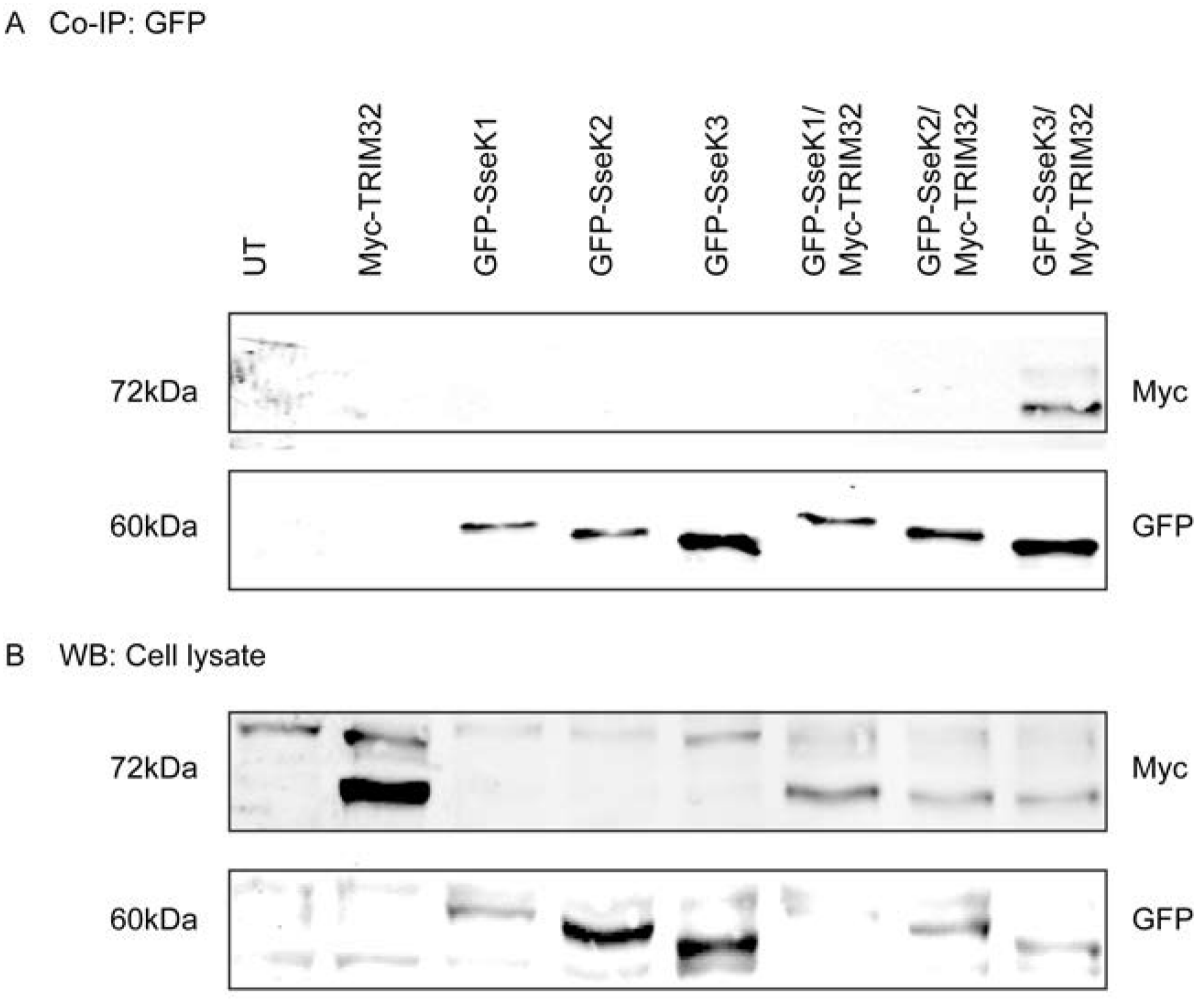
GFP-SseK3 preferentially interacts with non-ubiquitinated TRIM32. Sub-confluent A431 cells were transiently transfected with plasmids encoding GFP-SseK1, GFP-SseK2, GFP-SseK3 or Myc-TRIM32 alone, or both GFP-SseK and Myc-TRIM32. 16–18 h post transfection, cells were washed with chilled PBS and lysed on ice using TK lysis buffer. Equal amount of cell lysates were used for the immunoprecipitation using GFP-nanotrap beads. Immunoprecipitated complexes **(A)** and cell lysates **(B)** were resuspended in SDS sample loading buffer and boiled for 5 min before resolved by SDS-PAGE, and transferred onto Immobilon-FL membrane. After blocking in Odyssey blocking buffer, membranes were incubated with primary antibodies against GFP and Myc, followed by IRDye conjugated secondary antibodies. Fluorescence was detected by Li-COR Odyssey infrared imaging system. Untransfected cells were used as a negative control. Representative images from three independent experiments are shown.

To further validate the interaction between SseK3 and TRIM32, as well as determine whether ubiquitinated TRIM32 was able to interact with SseK3, GFP-SseK1, GFP-SseK2, GFP-Ssek3 and Myc-TRIM32 were co-expressed ectopically in A431 cells. GFP-SseK1, GFP-SseK2 and GFP-SseK3 was immunoprecipitated from cell lysates as described above and Myc-TRIM32 was detected by immunoblotting with an anti-Myc antibody. As shown previously, the non-ubiquitinated form of TRIM32 represents the main form of Myc-TRIM32 detected in the whole cell lysate, whereas mono-ubiquitinated Myc-TRIM32 protein was also detected but at much lower levels (Fig 2). We found GFP-SseK3 co-immunoprecipitated with non-ubiquitinated Myc-TRIM32, but also with mono-ubiquitinated Myc-TRIM32 (Fig 2). Similarly when Myc-TRIM32 was immunoprecipitated from cells co-expressing Myc-TRIM32 and EGFP-SseK1, EGFP-SseK2 or EGFP-SseK3, only EGFP-SseK3 co-immunoprecipitated with Myc-TRIM32 (S2 Fig). Overall our data suggested that SseK3 selectively interacted with TRIM32 compared to other SseK proteins. While SseK3 appears to interact predominantly with the non-ubiquitinated form of TRIM32 (Fig 1), it does have the capacity to interact with monoubiquitinated TRIM32 (Fig 2).

### TRIM32 is not modified with GlcNAc by SseK3

Previous studies have shown NleB1 possesses GlcNAc transferase activity, and modifies host protein targets such as FADD, resulting in reduced cell death during EHEC/EPEC infection [11, 12]. Overexpression of NleB also inhibits activation of an NF-κB dependent luciferase reporter in response to TNF [14]. Mutation of the catalytic site in NleB (D221A and D223A) results in loss of GlcNAc transferase activity and the ability of NleB to block death receptor signaling. The DxD motif is highly conserved in the SseK family members and SseK1 was shown to GlcNAcylate TRADD resulting in reduced NF-κB dependent luciferase activity in response to TNF [11]. Therefore, we determined whether SseK3 possessed GlcNAc transferase activity towards TRIM32 and influenced NF-κB activation.

**Fig 3:**
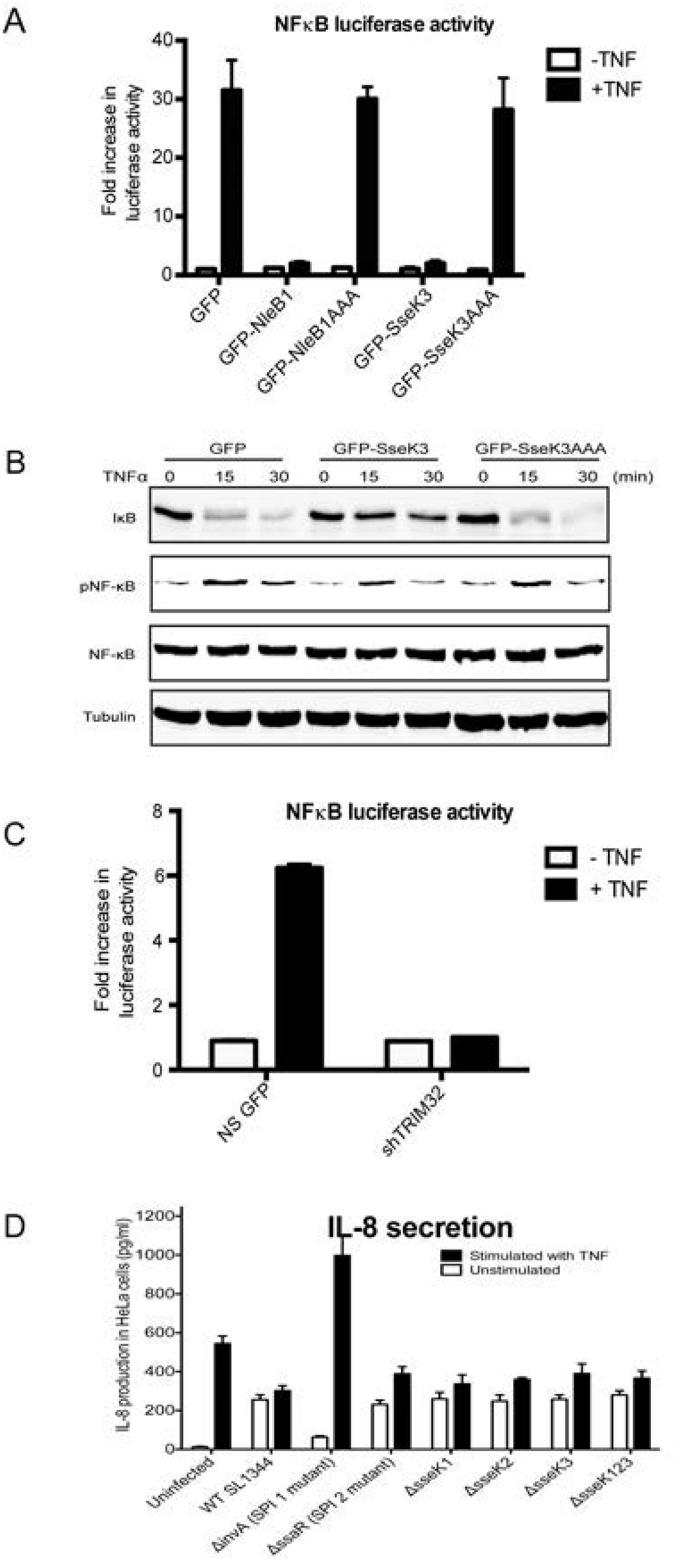
GFP-SseK3 inhibits TNFα stimulated NFκB luciferase activity. **(A)** Sub-confluent Hela cells were transiently co-transfected with plasmids encoding GFP-SseK3, GFP-SseK3AAA, GFP-NleB1 or GFP-NleB1AAA plasmid together with pNF-κB-Luc plasmid and the control pRL-TK plasmid. 16–18 h post transfection, cells were stimulated with 20 ng/ml of TNFα for 16 h. Following, the cells were harvested and analysed by the dual luciferase reporter assay according to the manufacturer’s instructions using a FluoStar Omega plate reader. The firefly luciferase activity of each sample was normalized to the *Renilla* luciferase activity. Graph represents the fold increase of NF-κB luciferase activity from three independent experiments. **(B)** HeLa were transiently transfected with GFP, GFP-SseK3 or GFP-SseK3AAA plasmids. 16–18 h post transfection, cells were stimulated with 20ng/ml of TNFα for 15 and 30 min. Following, the cells were harvested in TK lysis buffer. After BCA protein quantitation, equal amounts of protein samples were subjected for SDS-PAGE and western blots. Membranes were incubated with anti-IκB, anti-phospho Ser536 NFκB p65, or anti-NFκB p65 antibodies, whereas anti-tubulin antibody was included as a loading control. After incubation with IRDye conjugated fluorescence secondary antibodies, fluorescence intensities were detected and scanned by using Li-COR Odyssey infrared imaging system. **(C)** HeLa non-silence GFP control cells and TRIM32 knockdown cells were transiently transfected with pNF-kB-Luc plasmid, followed by the stimulation with 20 ng/ml of TNFα for 16 h. Cells were harvested for Luciferase reporter assay according to the manufacturer’s instructions. **(D)** HeLa cells were infected with *Salmonella* strains for 1 h. After culture medium was replaced by the medium containing 100 μg/ml gentamycin for 1 h, cells were further grown in the medium containing 10 μg/ml gentamycin with untreated or 20 ng/ml TNFα stimulation for additional 6 hrs. The supernatants were collected to measure IL-8 secretion using the Quantikine Human IL-8 Immunoassay according to the manufacturer’s instructions.

The residues within EGFP-SseK3 that align to the catalytically active site within NleB1 were identified by sequence alignment. These residues were then mutated into alanine (D226A, D228A) within SseK3 (EGFP-SseK3AAA). Firstly, we determined if ectopic overexpression of SseK3 affected NF-κB dependent luciferase activity. EGFP, EGFP-SseK3 or EGFP-SseK3AAA expressing HeLa cells were co-transfected to express the NF-κB luciferase reporter and treated with 20 ng/ml TNF for 16 h. EGFP-NleB1 expressing HeLa cells were used as a positive control. TNF stimulation led to ~30-fold increase of NF-κB dependent luciferase activity, compared to unstimulated cells, in EGFP transfected cells. Consistent with previous study [12], NleB1 overexpression dampened TNF-stimulated NF-κB activity, whereas expression of NleB1AAA mutant did not block NF-κB activity by TNF. Similar to NleB1, SseK3 overexpression impaired TNF-induced NF-κB dependent luciferase activity (Fig 3). In contrast, EGFP-SseK3AAA was not able to inhibit TNF-induced NF-κB activity. Furthermore, similar to NleB1, ectopic expression of SseK3 partially inhibited TNF-induced IκB degradation, leading to the decreased NF-κB p65 phosphorylation at Ser536 residue, whereas the SseK3AAA mutant did not (Fig 3), suggesting that this activity depended on the DxD motif.

To determine the role of TRIM32 in NF-κB activation, TRIM32 expression was reduced by lentiviral shRNA knock-down. In TRIM32 knock-down cells, activation of the NF-κB dependent luciferase reporter by TNF was inhibited compared to non-silence control (Fig 3), suggesting that TRIM32 may regulates the host inflammatory response. This may implicate SseK3 binding to TRIM32 in the inhibition of NF-κB activation. However, deletion of *sseK3* or even *sseK1/2/3* from strain SL1344 had no impact on the production of the NF-κB dependent cytokine, IL-8, during *Salmonella* infection of HeLa cells, even upon exogenous stimulation with TNF (Fig 3). Hence the effect of SseK3 on the inflammatory response is uncertain, at least during *in vitro* infection.

**Fig 4:**
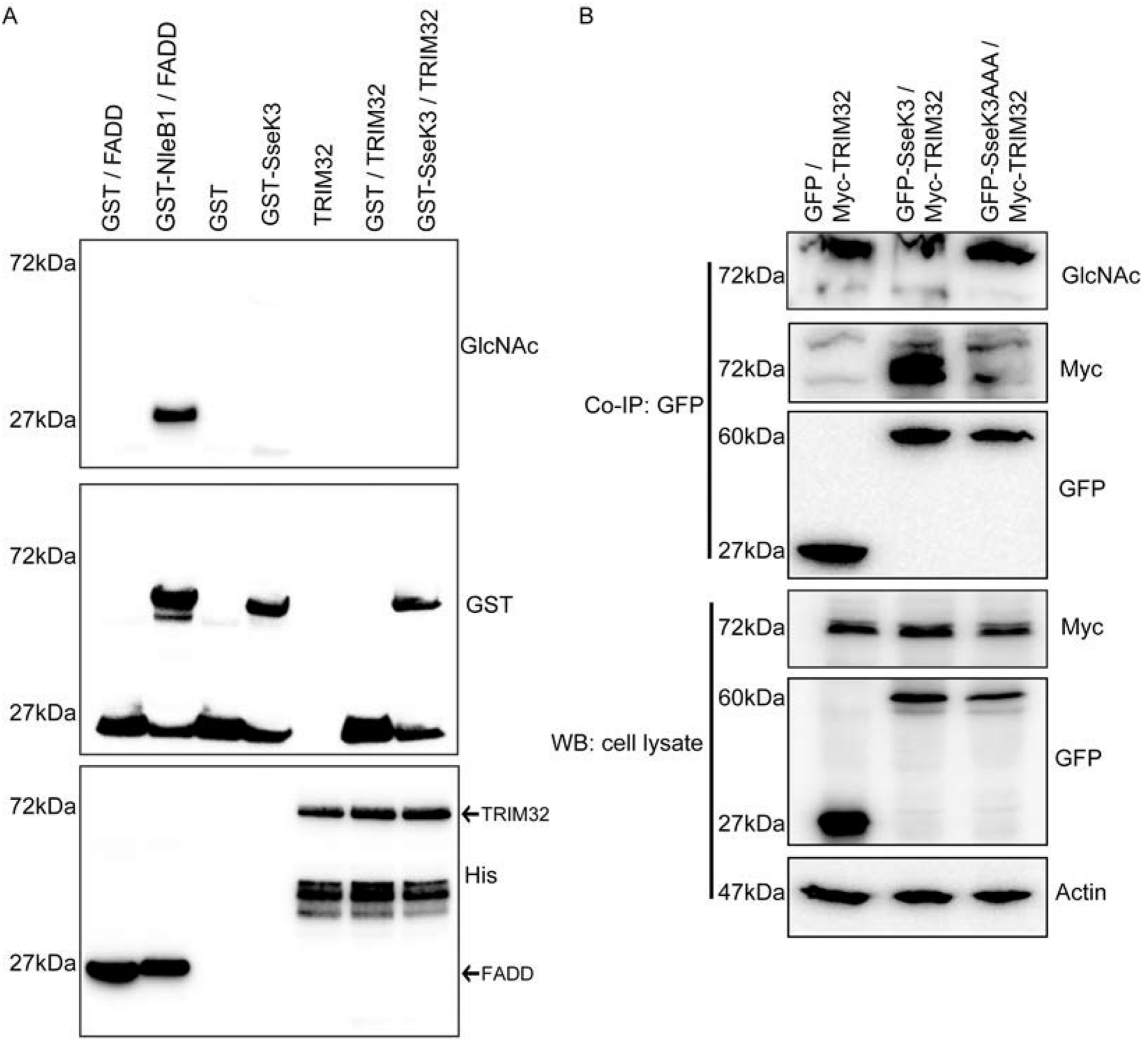
SseK3 does not GlcNAcylate TRIM32. **(A)** 2 μg of purified recombinant GST or GST-SseK3 were incubated with His-TRIM32 at 37°C for 4 hrs in the presence of 1 mM UDP-GLcNAc. In parallel, GST-NleB1 was incubated with His-FADD in the presence of UDP-GLcNAc as a positive control for the assay. Following, samples were boiled and subjected to SDS-PAGE and western blot, and probed with anti-GlcNAc, anti-GST and anti-His antibodies. The membranes were further incubated with HRP conjugated secondary antibodies before signals detected by using ECL systems. Representative images from three independent experiments are shown. **(B)** Sub-confluent HEK293T cells were co-transfected with GFP, GFP-SseK3 or GFP-SseKAAA together with Myc-TRIM32 plasmid. 16–18 h post transfection, the transfected cells were harvested in lysis buffer, and the equal amount of pre-cleared cell lysates were used for immunoprecipitation using GFP-nanotrap beads. Input whole cell lysates and immunoprecipitated samples were boiled for 5 mins and subjected to SDS-PAGE and western blots. Membranes were probed with anti-GlcNAc, anti-GFP, or anti-myc antibodies, whereas anti-actin was used as a loading control. The membranes were further probed with HRP conjugated secondary antibodies before developing with ECL system. Representative images from three independent experiments are shown.

The GlcNAc transferase activity of NleB1 modifies death domain containing proteins such as FADD and TRADD, which disrupts the formation of death receptor signaling complexes. Despite this, NleB1 has no impact on the inhibition of pro-inflammatory cytokine production such as IL-8 [12]. To test whether the impaired NF-κB activity by SseK3 is due to TRIM32 being a substrate for SseK3, *in vitro* N-acetyl-D-glucosamine transferase assay was performed. Consistent with previous findings, recombinant purified GST-NleB1 exhibited GlcNAc transferase towards His-FADD (Fig 4) [12]. Under the same conditions His-TRIM32, GST and GST-SseK3 proteins were purified from bacterial expression systems and incubated in the presence of UDP-GlcNAc. However, immunoblotting with antibodies specific to GlcNAc did not detect any GlcNAcylation of His-TRIM32 by SseK3 (Fig 4). However, the His-TRIM32 recombinant protein appeared to be poorly folded based on gel filtration chromatography and readily precipitated from solution.

The GlcNAc transferase activity of SseK3 was tested further in a cell model. Myc-tagged TRIM32 was co-expressed with GFP-SseK3 or GFP-SseK3AAA in HEK293 cells. Immunoprecipitation then was performed by using GFP-nanotrap beads. However, no GlcNAcylation was detected for myc-TRIM32 when immunoblotting with anti-GlcNAc antibody (Fig 4). Hence, either TRIM32 is not GlcNAcylated by SseK3 or the GlcNAcylation level on TRIM32 was below our detection levels. In addition, we did not detect self-GlcNAcylation activity towards SseK3 (data not shown). The anti-GlcNAc antibody is raised to O-linked GlcNAc [29] and while this recognises N-linked arginine modification by NleB, the affinity of the antibodies for N-linked modification of TRIM32 might fall below the cut-off for detection in this instance. Alternatively, SseK3 may not be a GlcNAc transferase, as the DxD motif is present in a range of glycosyltransferases, where it functions as the carbohydrate binding motif. While the sequence homology of SseK3 indicates it will likely be a glycosyltransferases SseK3 might possess other post-translational modifications activity towards its substrates. For example, it has been shown that many bacteria secretion effectors have AMPylation activities towards host GTPase [30]. DrrA, a Legionella virulent factor, can AMPylate Rab1b at Tyr77 residue, thereby preventing GTP hydrolysis and making Rab1b constitutively active [31]. DrrA contains an adenylyl transferase domain, which has a conserved G-X-D-X-D motif and those asparatate residues are essential for the AMPylation activity [30, 31]. Given that the D-X-D sequence is presented on SseK3, hence, the PTMs on SseK3, including AMPylation, need to be elucidated in future studies.

**Fig 5:**
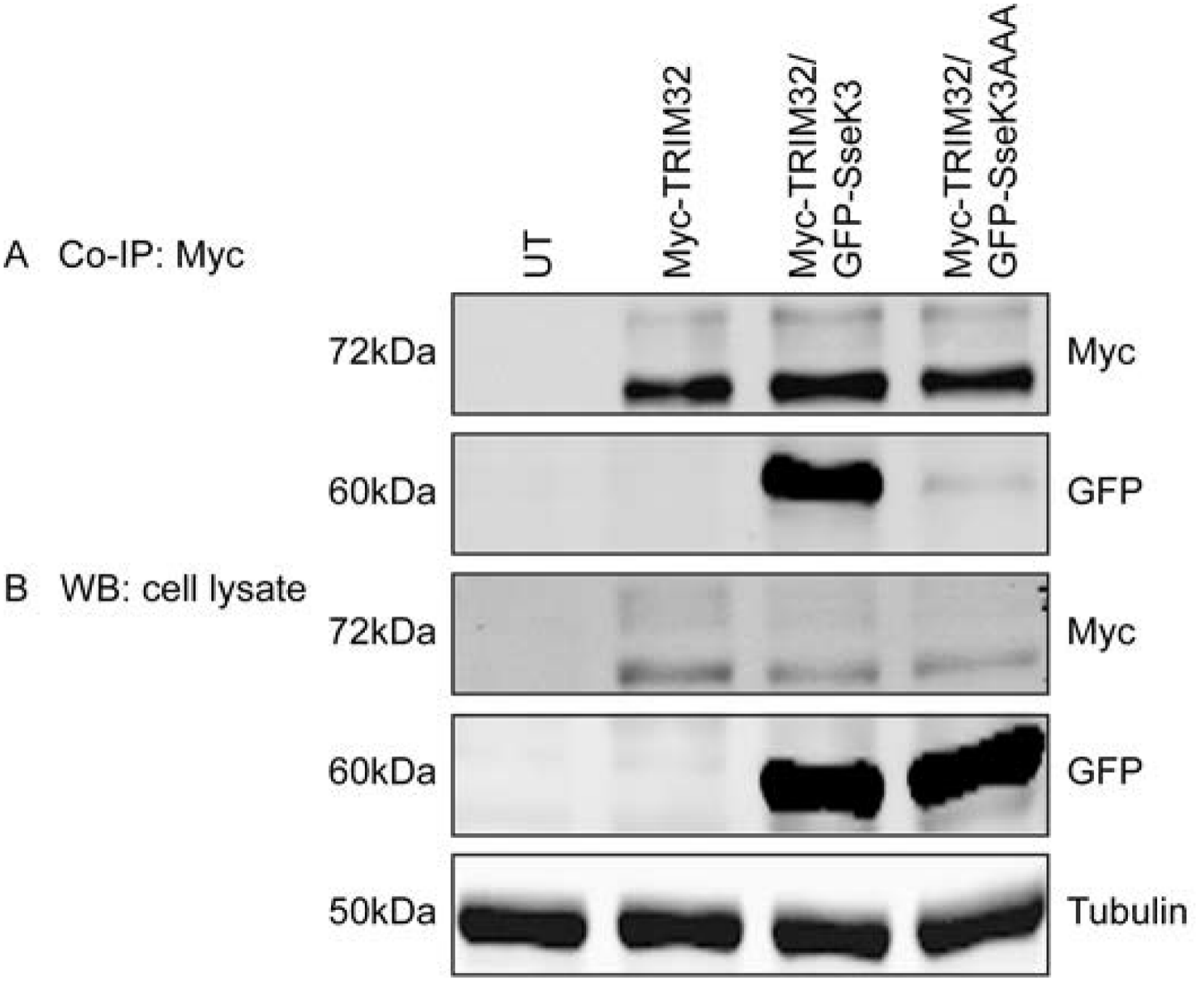
SseK3AAA mutant has reduced affinity for Myc-TRIM32. Sub-confluent A431 cells were transiently co-transfected with plasmids encoding Myc-TRIM32 and GFP-SseK3 or GFP-SseK3AAA. Untransfected cells were included as a negative control. 16–18 h post transfection, cells were then washed and harvested in TK lysis buffer. Equal amounts of pre-cleared cell lysates were used for immunoprecipitation using mouse monoclonal anti-Myc antibody coupling with Protein G agarose beads. Immunoprecipitated proteins **(A)** and whole cell lysates **(B)** were boiled for 5 min in SDS sample loading buffer and resolved by SDS-PAGE/ western blots. Membranes were incubated with anti-GFP or anti-Myc antibodies, whereas anti-tubulin antibody was included as a loading control. After incubation with IRDye conjugated fluorescence secondary antibodies, fluorescence intensities were detected and scanned by using Li-COR Odyssey infrared imaging system. Representative images from three independent experiments are shown.

In addition, we observed the strong reduction of Myc-TRIM32 protein level from GFP-SseK3AAA immunoprecipitated samples (Fig 4). To confirm this, Myc-TRIM32 was immunoprecipitated from cells co-transfected with Myc-TRIM32 and either GFP-SseK3 or GFP-SseK3AAA (Fig 5). GFP-SseK3 co-immunoprecipitated with Myc-TRIM32 with little GFP-SseK3AAA detected despite similar levels of protein expression for GFP-SseK3 and GFP-SseK3AAA (Fig 5). Therefore the DxD motif of SseK3 was critical for the interaction between TRIM32 and SseK3. This is similar to NleB, as the NleBAAA mutant is not able to bind to the death domain containing proteins, such as TRADD [11] but differs from, reports that the NleB1AAA mutant is still able to bind to substrates such as FADD [12].

### Ectopic expression of SseK3 does not alter the ubiquitination of TRIM32

**Fig 6:**
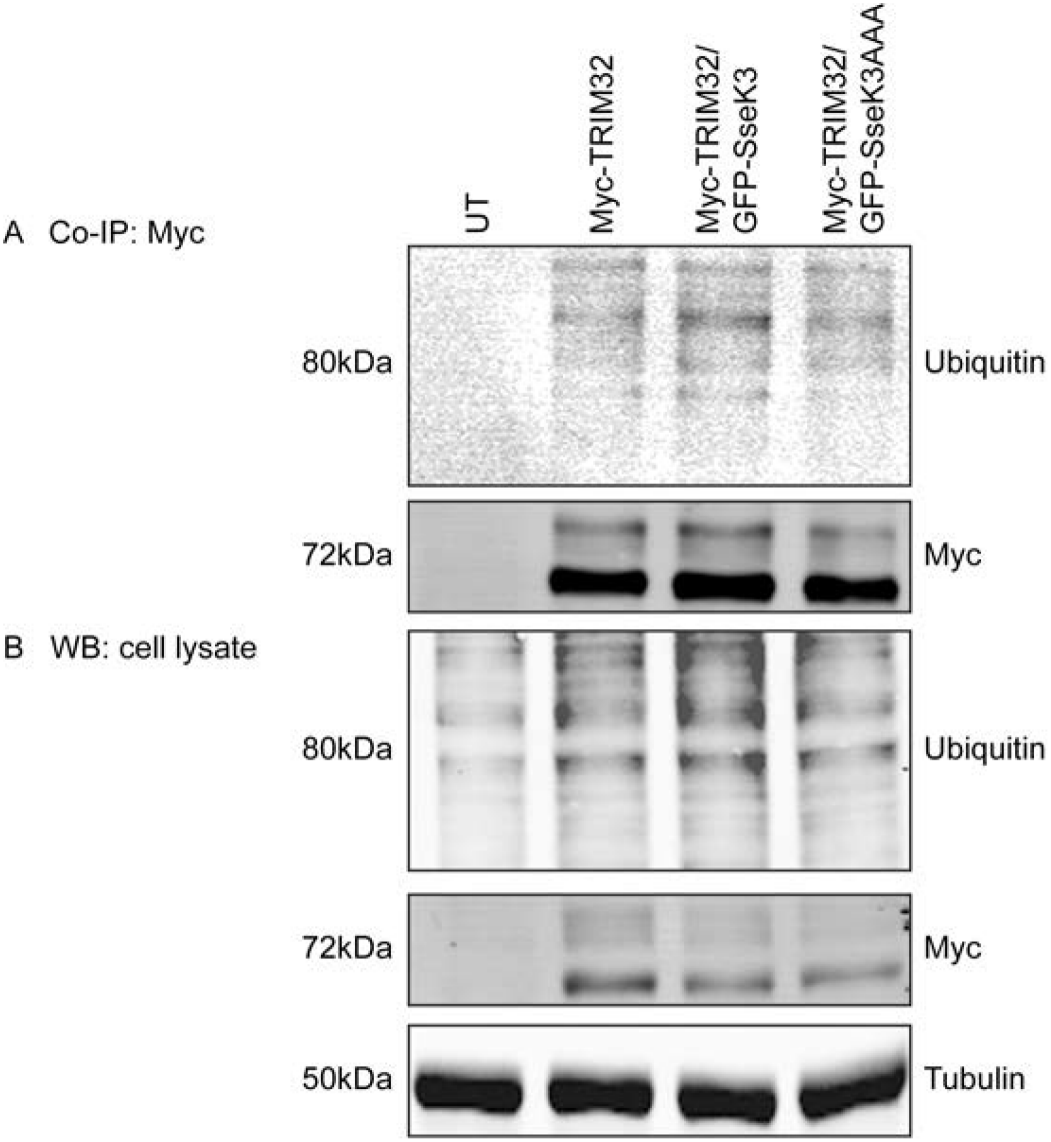
Ectopic expression of SseK3 does not alter the ubiquitination of TRIM32. Sub-confluent A431 cells were transiently co-transfected with plasmids encoding Myc-TRIM32 and GFP-SseK3 or GFP-SseK3AAA. Untransfected cells were included as a negative control. 16–18 hrs post transfections, cells were then washed and harvested in TK lysis buffer. Equal amounts of pre-cleared cell lysates were used for immunoprecipitation using mouse monoclonal anti-Myc antibody coupling with Protein G agarose beads. Immunoprecipitated proteins **(A)** and whole cell lysates **(B)** were boiled for 5 min in SDS sample loading buffer and resolved by SDS-PAGE/ western blots. Membranes were incubated with anti-ubiquitin or anti-Myc antibodies, whereas anti-tubulin antibody was included as a loading control. After the incubation with IRDye conjugated fluorescence secondary antibodies, fluorescence intensities were detected and scanned by using Li-COR Odyssey infrared imaging system. Represented images from three independent experiments were shown. The asterix (*) indicates 80 kDa of mono-ubiquitinated TRIM32.

TRIM32, like other E3 ubiquitin ligases, is capable of regulating its own activity by auto-ubiquitination [32, 33]. To determine if the interaction between TRIM32 and SseK3 modulated the TRIM32 auto-ubiquitination or led to ubiquitination of SseK3, Myc-TRIM32 was co-transfected with GFP-SseK3 or GFP-SseK3AAA. Immunoblotting of cell lysates using a monoclonal anti-ubiquitin antibody showed that TRIM32 overexpression increased the ubiquitin levels within cells, compared to untransfected cells (Fig 6). Co-transfection of GFP-SseK3 or GFP-SseK3AAA with TRIM32 did not modulate ubiquitination induced by TRIM32 overexpression (Fig 6). Immunoprecipitated Myc-TRIM32 contained both mono- and poly-ubiquitin modifications but no change of the ubiquitin levels on was observed in cells co-transfected with GFP-SseK3 or GFP-SseK3AAA (Fig 6). In addition when GFP-SseK3 or GFP-SseK3AAA was immunoprecipitated from cells co-transfected with Myc-TRIM32, there were no detectable levels of ubiquitin on GFP-SseK3 or GFP-SseK3AAA (data not shown). Overall our results indicated that the auto-ubiquitination activity of TRIM32 was not altered upon co-expression with SseK3, and that SseK3 was not a substrate for ubiquitination by TRIM32.

### Subcellular localisation of SseK3 and TRIM32

**Fig 7:**
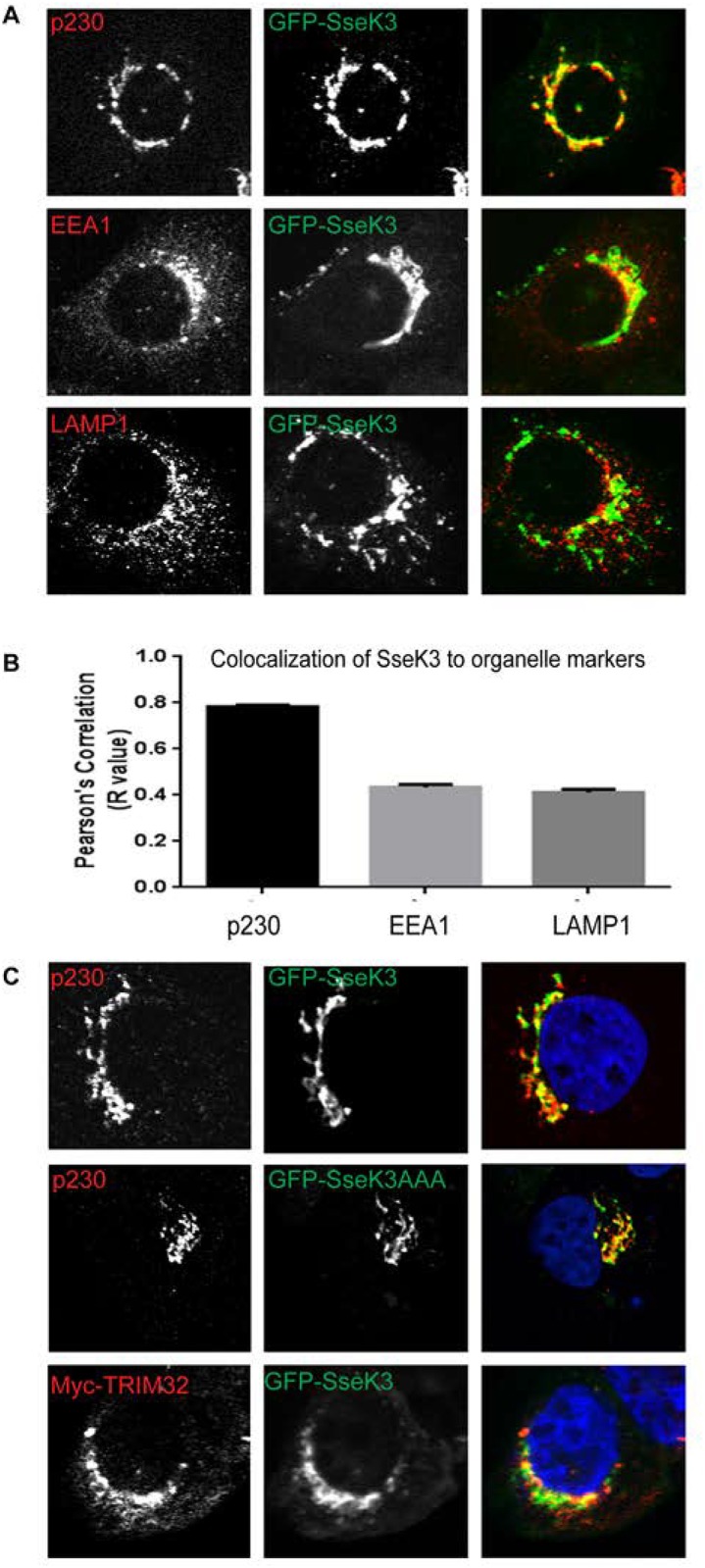
GFP-SseK3 localises to the trans-Golgi network. **(A)** Sub-confluent A431 cells grown on coverslips were transiently transfected with a plasmid encoding GFP-SseK3. 16–18 h post transfection, cells were fixed in 4% PFA and subjected to indirect immunofluorescence by staining with anti-p230, anti-EEA1 or anti-LAMP1 antibodies, followed by Alexa Fluor conjugated secondary antibodies. Images were captured using a Zeiss LSM 510 confocal laser-scanning microscope under 63x Oil objective. **(B)** Quantification of co-localisation between GFP-SseK3 and endogenous markers. Graph represents the mean Pearson’s Correlation (R value) of 100 cells randomly selected from 20 individual images. Error bars represent SEM. **(C)** A431 cells were transfected with plasmids encoding GFP-SseK3 or GFP-SseK3AAA alone, or Myc-TRIM32 and GFP-SseK3 together, and subjected to indirect immunofluorescence using anti-p230 and anti-Myc antibodies, followed by Alexa Fluor conjugated secondary antibodies. Images were captured using a Zeiss LSM 510 confocal laser-scanning microscope under 63x Oil objective. Scale bar 10μm.

To determine the subcellular localisation of SseK3, GFP-SseK3 was transiently transfected into A431 cells, observed by immunofluorescence microscopy. GFP-SseK3 localised to numerous discrete punctate perinuclear structures within the cell (Fig 7). SseK3 showed obvious co-localisation with p230, a marker of the trans-Golgi network (Fig 7) but showed little co-localisation with early endosomes (EEA1) and lysosomes (LAMP1). Quantification of co-localisation was performed using ImageJ software. Pearson’s Correlation analysis found GFP-SseK3 had a high level of co-localisation with p230 compared to the other endogenous markers examined (Fig 7). Similar to GFP-SseK3, ectopically expression of GFP-SseL3AAA also co-localized with p230 (Fig 7), indicating that subcellular localization of SseK3 is not altered when putative DxD motif is mutated. TRIM32 has been localized to multiple subcellular localizations, including mitochondria, ER and nucleus [18]. When GFP-SseK3 was co-expressed with Myc-TRIM32, strong co-localisation was observed between SseK3 and TRIM32 on punctate perinuclear structures (Fig 7). Overexpression of Myc-TRIM32 had no effect on GFP-SseK3 co-localization with p230, and *vice versa*, GFP-SseK3 overexpression did not affect the subcellular localization of TRIM32.

The subcellular localization of GFP-SseK3 during *Salmonella* infection was examined to determine if it was recruited to SCV’s. A431 cells were transfected with GFP-SseK3 before being infected with SL1344 expressing RFP at MOI = 1 for up to 20 h before fixation and analysis by immunofluorescence microscopy. During SL1344 infection, the subcellular localization of GFP-SseK3 was not changed and the effector was not recruited to the SCV or *Salmonella* induced filaments (SIF’s) observed at 20 h post infection (S3 Fig).

The ability of *Salmonella* effectors to interact with host proteins elicits multiple outcomes, such as altering host protein trafficking or innate immunity response, during infection. For example, the SPI1 *Salmonella* effector, SopB, is required for the recruitment of host proteins Rab5 and Vps34 to the SCV, which in turn promote the formation of phosphatidylinositol 3-phosphate on the outer surface of the SCV, which assists maturation of the bacterium’s niche within host cells [34]. The SPI-II *Salmonella* effector, SifA, interacts with SifA and kinesin-interacting protein (SKIP), which is essential for the formation of *Salmonella* induced filaments (SIFs). In contrast to these effectors, SseK3 has no known role in the formation of *Salmonella* induced structures or host protein trafficking to establish a niche for *Salmonella* survival. Other effectors have a role in the regulation of host innate response. For examples, AvrA posses acetyltransferase activity towards MEKK4/7 to dampen JNK activation; whereas host protein SGT1 binds to SspH2 to increase the E3 ubiquitin ligase activity of SspH2, which in turn ubiquitinates and enhances Nod1 activity to modulate host innate response [35]. Furthermore, the SPI2 effector, GogB, targets the host SCF E3 ubiquitin ligase to limit NF-κB activation [13]. Here we observed that over expression of SseK3 also modulated the NF-κB signalling pathway and this required the DxD motif necessary for GlcNAc transferase activity in related bacterial effectors. However, deletion of *sseK3* from *Salmonella* had no effect on IL-8 production during infection. This is similar to NleB, which despite suppression of an NF-κB dependent luciferase reporter when expressed ectopically, has no impact on the suppression of IL-8 production during EPEC infection [12].

We identified TRIM32 as a potential binding partner of SseK3 but we could not detect any GlcNAc modification of TRIM32 by SseK3, suggesting that the physical interaction of the two proteins may promote localisation rather than inhibition of function. Nevertheless, the knock down of TRIM32 activity using RNAi suppressed TNF-induced NF-κB signalling in non-infected cells showing that TRIM32 has a role in modulation of the NF-κB pathway. It remains possible that SseK3 targets an as yet unidentified host protein for GlcNAcylation as to date we have not been able to detect any interaction or GlcNAc modification of death domain containing proteins by SseK3 (data not shown). Hence, we postulate that the enzymatic function of SseK3 is similar to NleB but the host target proteins are different.

## Conclusion

From this study we found that the *Salmonella* effector SseK3 reduces NF-κB activity and that the DxD motif within the GlcNAc transferase domain of SseK3 is essential for this activity. In addition we discovered a novel interaction between SseK3 and the host protein, TRIM32, that while dependent on the DxD motif of SseK3 did not appear to involve the direct modification of TRIM32 with GlcNAc. These findings give further insight into host-*Salmonella* interactions, the characterisation of which is essential in order to understand the factors that contribute to the pathogenesis of *Salmonella* as well as other enteric bacterial pathogens.

## Supporting Information

S1 Fig Identification of specific protein binds to GFP-SseK3. Sub-confluent HEK293 cells were transiently transfected with plasmids encoding GFP alone, GFP-SseK1, GFP-SseK2 or GFP-SseK3. 16–18 h post transfection, cells were washed with chilled PBS and lysed on ice using TK lysis buffer. Equal amounts of pre-cleared cell lysates were used for immunoprecipitation with GFP nanotrap beads. Immunoprecipitated proteins were boiled for 5 min in SDS sample loading buffer and resolved by SDS-PAGE, followed by Colloidal Coomassie Blue. The excised region of the gel containing proteins specifically interacting with SseK3 is indicated by arrow.
(PDF)

S2 Fig Ectopic Myc-TRIM32 binds to GFP-SseK3. Sub-confluent A431 cells were transiently transfected with plasmids encoding GFP-SseK1, GFP-SseK2, GFP-SseK3, Myc-TRIM32 alone, or Myc-TRIM32 together with GFP-SseK1, GFP-SseK2 or GFP-SseK3 plasmids. 16–18 h post transfection, cells were washed with chilled PBS and lysed on ice using TK lysis buffer. Equal amounts of pre-cleared cell lysates were used for immunoprecipitation using mouse monoclonal anti-Myc antibody coupling with Protein G agarose beads. Immunoprecipitated proteins **(A)** and whole cell lysates **(B)** were boiled for 5 min in SDS sample loading buffer and resolved by SDS-PAGE/ western blots. Membranes were incubated with anti-GFP and anti-Myc antibodies. After the incubation with IRDye conjugated fluorescence secondary antibodies, fluorescence intensities were detected and scanned by using Li-COR Odyssey infrared imaging system. Represented images from three independent experiments were shown.
(PDF)

S3 Fig Subcellular localization of GFP-SseK3 during Salmonella infection. Sub-confluent A431 cells were grown on coverslips and transiently transfected with a plasmid encoding GFP-SseK3. 16–18 h post transfection, transfected cells were infected with RFP-SL1344 at a multiplicity of infection (MOI) of 1 using gentamicin protection assay. At each time point post infection, cells were fixed in 4% PFA. After mounting to slides, the images were captured using a Zeiss LSM510 Inverted Scanning Laser confocal microscope at 63x magnification. Scale bar 10μm.
(PDF)
