# Supplementary figures and images for: SseK3 Is a Salmonella Effector That Binds TRIM32 and Modulates the Host’s NF-κB Signalling Activity
Source: PLoS One. 2015 Sep 22;10(9):e0138529. doi: 10.1371/journal.pone.0138529 (PMC4579058; doi:10.1371/journal.pone.0138529)

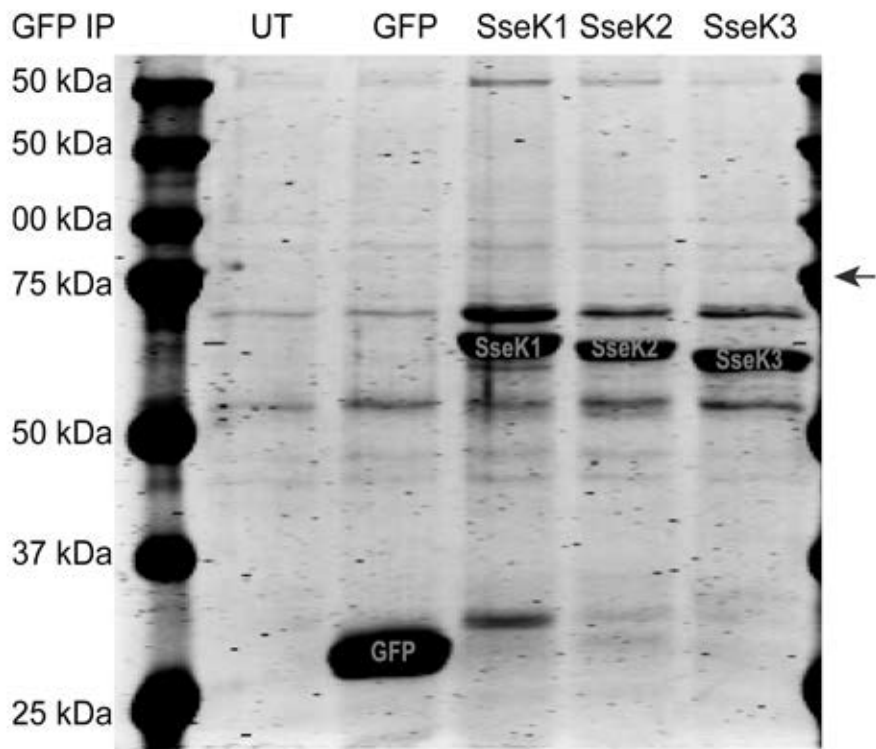

Supplementary Figure 1

Supplement: S1 Fig — Sub-confluent HEK293 cells were transiently transfected with plasmids encoding GFP alone, GFP-SseK1, GFP-SseK2 or GFP-SseK3. 16–18 h post transfection, cells were washed with chilled PBS and lysed on ice using TK lysis buffer. Equal amounts of pre-cleared cell lysates were used for immunoprecipitation with GFP nanotrap beads. Immunoprecipitated proteins were boiled for 5 min in SDS sample loading buffer and resolved by SDS-PAGE, followed by Colloidal Coomassie Blue. The excised region of the gel containing proteins specifically interacting with SseK3 is indicated by arrow. (PDF) [file pone.0138529.s001.pdf]

A Co-IP: Myc

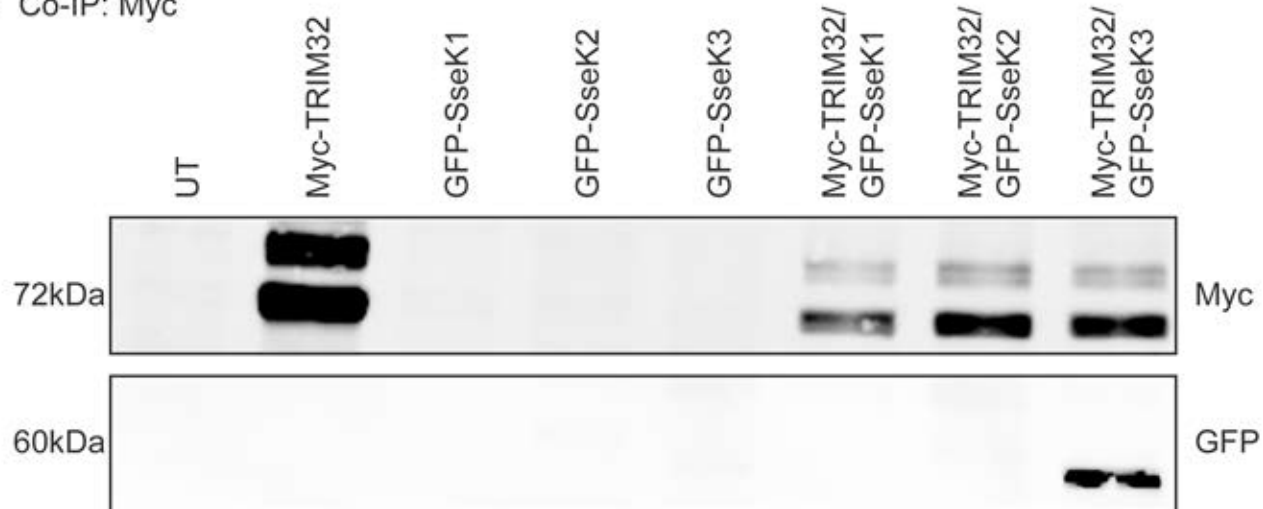

B WB: cell lysate

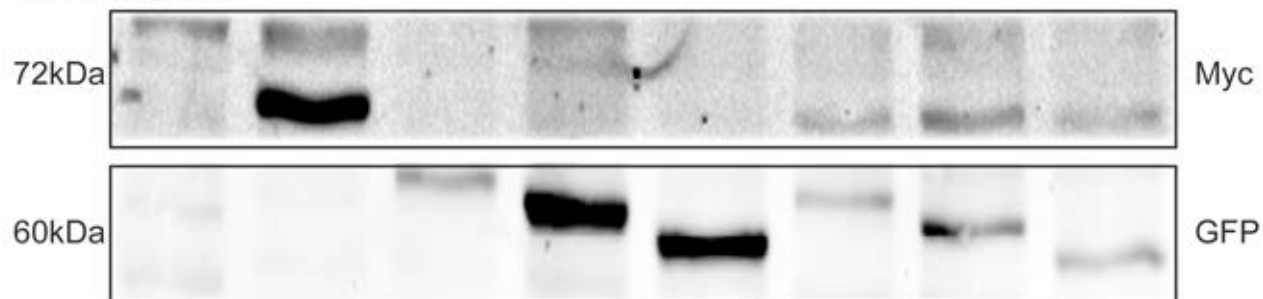

Supplementary Figure 2

Supplement: S2 Fig — Sub-confluent A431 cells were transiently transfected with plasmids encoding GFP-SseK1, GFP-SseK2, GFP-SseK3, Myc-TRIM32 alone, or Myc-TRIM32 together with GFP-SseK1, GFP-SseK2 or GFP-SseK3 plasmids. 16–18 h post transfection, cells were washed with chilled PBS and lysed on ice using TK lysis buffer. Equal amounts of pre-cleared cell lysates were used for immunoprecipitation using mouse monoclonal anti-Myc antibody coupling with Protein G agarose beads. Immunoprecipitated proteins (A) and whole cell lysates (B) were boiled for 5 min in SDS sample loading buffer and resolved by SDS-PAGE/ western blots. Membranes were incubated with anti-GFP and anti-Myc antibodies. After the incubation with IRDye conjugated fluorescence secondary antibodies, fluorescence intensities were detected and scanned by using Li-COR Odyssey infrared imaging system. Represented images from three independent experiments were shown. (PDF) [file pone.0138529.s002.pdf]

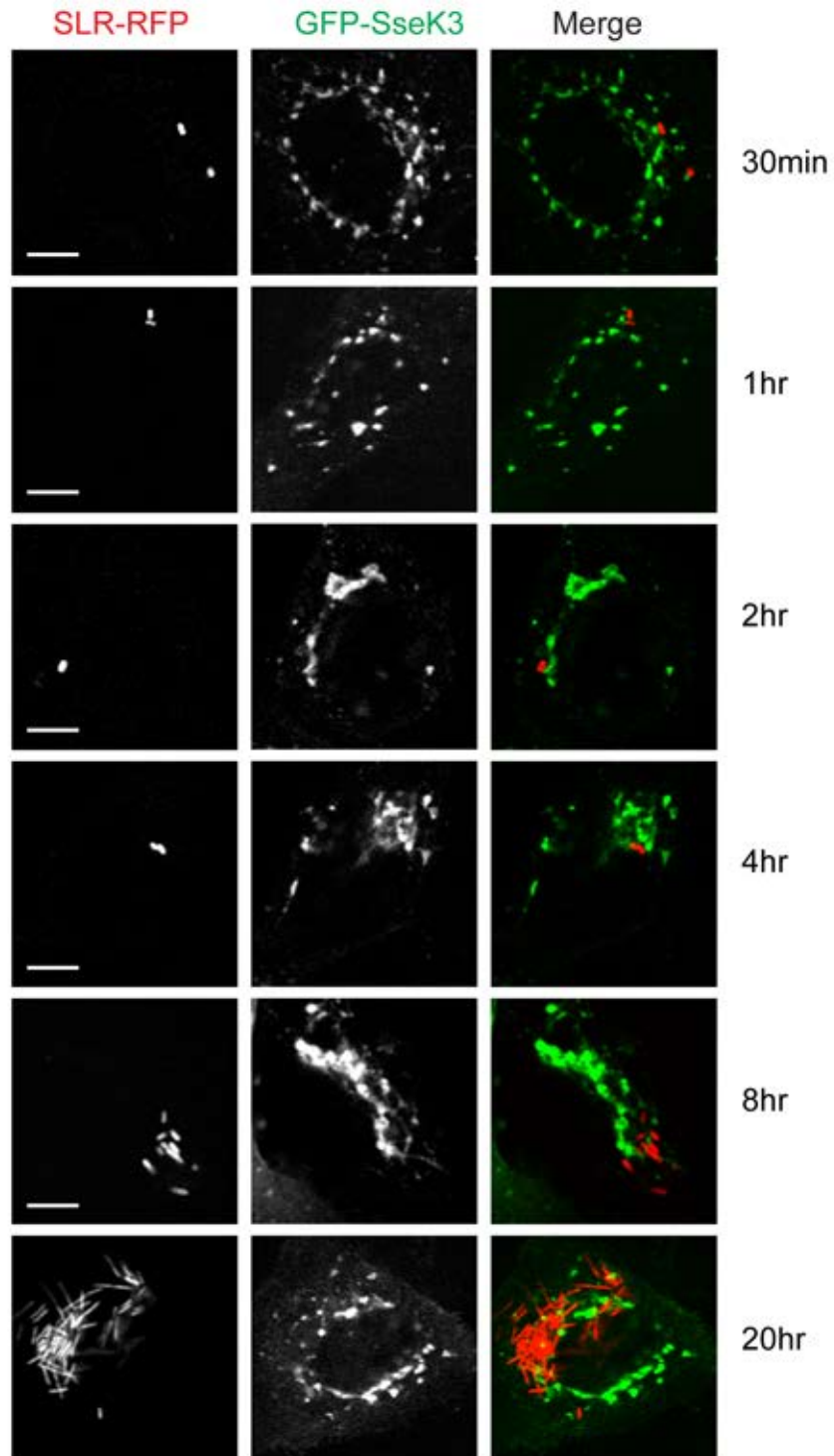

Supplementary Figure 5

Supplement: S3 Fig — Sub-confluent A431 cells were grown on coverslips and transiently transfected with a plasmid encoding GFP-SseK3. 16–18 h post transfection, transfected cells were infected with RFP-SL1344 at a multiplicity of infection (MOI) of 1 using gentamicin protection assay. At each time point post infection, cells were fixed in 4% PFA. After mounting to slides, the images were captured using a Zeiss LSM510 Inverted Scanning Laser confocal microscope at 63x magnification. Scale bar 10μm. (PDF) [file pone.0138529.s003.pdf]
